# Karyopherin alpha 2 is a novel prognostic marker and a potential therapeutic target for colon cancer

**DOI:** 10.1186/s13046-015-0261-3

**Published:** 2015-12-01

**Authors:** Yu Zhang, Meng Zhang, Fudong Yu, Su Lu, Huimin Sun, Huamei Tang, Zhihai Peng

**Affiliations:** Department of Pathology, Shanghai Jiaotong University Affiliated First People’s Hospital, Shanghai, 200080 People’s Republic of China; Department of General Surgery, Shanghai Jiaotong University Affiliated First People’s Hospital, Shanghai, 200080 People’s Republic of China

**Keywords:** Colorectal cancer, KPNA2, Prognosis, Proliferation

## Abstract

**Background:**

Karyopherin alpha 2 (KPNA2), a member of the karyopherin family, plays a vital role in carcinogenesis. Yet its role in colon cancer is poorly characterized. We sought to clarify the clinical significance of its dysregulated expression in human colon tumor specimens.

**Methods:**

We evaluated KPNA2 mRNA and protein expression by real-time polymerase chain reaction and Western blotting in 40 primary colon cancer tissues and paired adjacent normal colon mucosa specimens. KPNA2 protein expression in colon tissue microarray of tumor and normal tissue specimens and lymph node metastasis specimens obtained from 195 colon cancer patients were analyzed immunohistochemically. The effect of KPNA2 knockdown on carcinogenesis potential of human colon cancer cells was determined using Cell Counting Kit-8 (CCK8), colony formation, cell migration, and tumorigenesis in nude mice.

**Results:**

KPNA2 was expressed at higher levels in colon tumors and lymph node metastasis specimens than in normal tissues. Patients with KPNA2-positive tumors were significantly correlated with the American Joint Committee on Cancer (AJCC) stage (*p* = 0.01), T-classification (*p* = 0.018), regional lymph node metastasis (*p* = 0.025), distant metastasis (*p* = 0.014), and differentiated degree (*p* = 0.001). KPNA2 was shown to be an independent prognostic indicator of disease-free survival (HR 1.681; 95 % CI: 1.170–2.416; *p* = 0.005) and overall survival (HR 2.770; 95 % CI: 1.314–5.837; *p* = 0.007) for patients with colon cancer. Knockdown of KPNA2 expression inhibited colon cancer cell proliferation, colony formation, and migration.

**Conclusion:**

KPNA2 might play an important role in colorectal carcinogenesis and functions as a novel prognostic indicator and a potential therapeutic target for colorectal cancer.

## Background

In China, the incidence of colon cancer is increasing rapidly [1]. Both environmental (diet) and genetic factors play key roles in its etiology. Despite improvements in surveillance and therapeutic approaches, there are approximately 1,000 000 cases of colon cancer reported annually, with over 600,000 deaths per year [2]. Surgery alone rarely leads to complete tumor resection because of recurrence, which is a major factor in the failure of cancer treatment. Consequently, it was important to develop a prognostic tool for colon cancer patients to predict the risk of recurrence after curative resection.

Tumorigenesis and tumor progression are associated with dysfunction of the nuclear transport machinery at the level of import and export receptors (karyopherins) [3]. Aberrant expression of nuclear transport factors may cause altered, mutation-independent, subcellular localization of oncogene or tumor suppressors. Karyopherins act as carrier proteins between the cytoplasm and the nucleus. They mediate the shuttling of macromolecules larger than about 40 kDa, termed nucleocytoplasmic transport [4].

KPNA2 is a member of the karyopherin α-family, also known as importin α-1 or RAG cohort 1, weighs about 58 kDa and consist of 529 amino acids [5]. In recent years, KPNA2 has emerged as a potential biomarker in multiple forms of cancer, including breast carcinoma [6], lung cancer [7], melanoma [8], hepatocellular carcinima [9], and gastric carcinoma [10]. Rachidi has also reported the KPNA2 as one of the poor prognostic markers in colon cancer [11]. In addition, the observation of high levels of KPNA2 in the serum of lung cancer patients [12] indicated that KPNA2 could be a convenient and quick detection index for prognosis of cancer patients. As such, KPNA2 has been proposed as a prognostic biomarker for some epithelial malignancies [6–10].

In this study we examined the expression pattern of KPNA2, and evaluated its association with clinicopathologic features in colon cancer. In addition, a series of *in vitro* and *in vivo* assays were used to explore the role of KPNA2 in carcinogenesis of colon cancer.

## Methods

### Human tissue specimens and patient information

A total of 195 colon cancer patients were recruited at the time of diagnosis from patients treated at general surgery departments of Shanghai Jiaotong University Affiliated First People’s Hospital between 2001 and 2003. Patients received neither chemotherapy nor radiotherapy before surgery. There were 83 men and 112 women with (mean age: 65.82 years; range: 22–95 years). The follow-up period was 9–89 months (median: 61 months).

Collected clinical data included information about tumor localization, TNM stage according to American Joint Committee on Cancer (AJCC), and tumor grade. Disease-free survival (DFS) and overall survival (OS) durations were defined as the interval from the initial surgery to clinically or radiologically proven recurrence/metastasis and from initial surgery to death, respectively. The diagnosis was confirmed by at least two pathologists who were blinded to the data.

Forty pairs of fresh colon cancer samples and matched normal mucosa were obtained from patients who had undergone radical colectomy without preoperative therapy. The samples were put immediately into RNA Keeper Tissue Stabilizer (Vazyme Biotech Co, Ltd, Jiangsu, China) during the operation, stored at 4 °C overnight, and then stored at −80 °C for long-term storage. All patients provided written informed consent before entering the study. This research was approved by the institutional review boards of Shanghai Jiaotong University Affiliated Shanghai First People’s Hospital Medical Center.

### TMA construction and immunohistochemistry

TMA construction was undertaken as reported previously [13]. The expression of KPNA2 was tested using standard immunohistochemical methods [14, 15]. The corresponding primary antibodies were used as follows, KPNA2 (1:500 dilution, Abcam, Cambridge, UK) and Ki-67 (1:50 dilution, Abcam).

Positive staining was scored by two independent researchers blinded to patient information according to the staining area and intensity. Staining intensity was graded as follows: 0 for no staining; 1, mild staining; 2, moderate staining; and 3, intense staining. The staining area was scored as follows: 0, no staining of cells, 1, 1–25 %; 2, 26–50 %; 3, 51–75 %; and 4, 76–100 %. The sum of intensity and extension was designated as the staining score, graded as follows: 0–2, negative expression; 3–4, weak positive expression; and 5–7 strong positive expression. The immunostaining for Ki-67 was scored as negative group (none or ≤ 10 % cells with positive nuclei) and positive group (>10 % cells with positive nuclei).

### RNA extraction and quantitative real-time polymerase chain reaction

Total RNA was isolated from tissues or cell cultures using TRIzol reagent (Invitrogen Life Technologies, Carlsbad, CA). After the RNA integrity and purity were checked, the first-strand cDNA was synthesized from 500 ng of total RNA using PrimeScript/tm RT Master Mix (Takara, Shiga, Japan). Quantitative real-time polymerase chain reaction (RT-PCR) was performed with 1 μl of cDNA and the SYBR Premix Ex Taq II (Takara) as recommended by the manufacturer instruction. The following primers were used for the RT- PCR:

KPNA2 sense, 5’-CGTCGCAGAATAGAGGTCAA-3’;

KPNA2 antisense, 5’-GCGGAGAAGTAGCATCAG-3’;

OCT4 sense, 5’-CGCAAGCCCTCATTTCAC-3’;

OCT4 antisense, 5’-CATCACCTCCACCACCTG-3’;

GAPDH sense, 5’-AGAAGGCTGGGGCTCATTTG-3’;

GAPDH antisense, 5’-AGGGGCCATCCACAGTCTTC-3’;

Cycling conditions were as follows: initial denaturation 2 min at 95 °C followed by 40 cycles of denaturation (30 s at 95 °C), annealing (30s at 60 °C), and elongation (1 min at 72 °C). The final extension step was for 5 min at 72 °C; GAPDH was used as an internal control. Each PCR product was run in triplicate. The relative KPNA2 mRNA expression was calculated by 2^-ΔΔCt^.

### Western blot analysis

Total protein was isolated from colon tumors and their adjacent normal tissues using RIPA lysis buffer with inhibitor phenylmethanesulfonyl fluoride (PMSF) and then quantified by the BCA assay kit (Beyotime Biotechnology, Jiangsu, China). Equivalent amounts of protein (30 μg) were separated on 10 % SDS-PAGE gel and then transferred to polyvinylidene difluoride membranes. The membranes were blocked in 5 % non-fat milk with 0.1 % Tween 20 for 1 h at room temperature, and then probed with primary rabbit polyclonal antibody against human protein KPNA2 (1:1000, Abcam), OCT4 (1:1000, Abcam) or β-actin (1:1000, Abcam), followed by incubation with goat anti-rabbit IgG-HRP (1:2000 Santa Cruz Biotechnology, Santa Cruz, CA). The anti-β-actin antibody was used as loading controls. The blots were detected by Immobilon Western Chemiluminescent HRP substrate (Millipore, Billerica, MA) according to the manufacturer’s instructions.

### Cell culture and reagent

Human colon cancer cell lines RKO, HCT-116, CaCo2, SW-620, SW-480, HCT-8, HT-29 and LoVo were obtained from the Type Culture Collection of the Chinese Academy of Science (Shanghai, China) and cultured in Dulbecco’s modified Eagle’s medium (DMEM, Hyclone, Logan, UT), supplemented with 10 % fetal bovine serum (FBS, Gibco, Melbourne, Australia) and 1 % penicillin-streptomycin (Gibico). All the cell lines were maintained in a moist atmosphere (5 % CO_2_) at 37 °C.

### KPNA2 knockdown plasmid construction and cell transfection

The short hairpin RNA (shRNA) plasmid for KPNA2 and the control-shRNA plasmid were purchased from GeneChem Company (Shanghai, China). The shRNA with the sequence (shRNA1)5’-TGACATTGTCAAAGGCATA-3’and (shRNA2)5’-CAGAUACCUGCUGGGCUAUUUCCUA-3’ were used as the effective shRNA in inhibiting KPNA2 expression. The non-target shRNA sequence 5’-TTCTCCGAACGTGTCACGT-3’ was employed as the negative control. RKO and HCT-116 cells were transfected with plasmids using Lipofectamine 2000 (Invitrogen) according to the manufacturer’s instructions. After 72 h of transfection, antibiotic selection (2 μg/ml puromycin) was selected for 5 days. KPNA2 colon expressions were confirmed by qPCR and Western blot analysis.

### Cell proliferation assay

The effect of KPNA2 knockdown on colon cancer cell (RKO and HCT-116) proliferation was evaluated by plating transfected cells (KPNA2-shRNA or negative-control-shRNA) in 96-well plates (5 × 10^3^cells/well) in replicates of six. At the appropriate time (24, −48, −72 h), the cells were incubated with CCK-8 (10 μl/well; Dojindo, Japan) for 2 h at 37 °C. Absorbance was evaluated at a wavelength of 450 nm. The results presented as mean ± SD.

### Cell colony formation assays

The treated colon cancer cells were suspended and then plated in six-well plates (500cells/well) in triplicates. The cells were maintained in a moist atmosphere (5 % CO_2_) at 37 °C for 14 days. Following fixation, the cells were stained by Giemsa solution. The colonies that were 50 μm in diameter or larger were counted and photographed.

### Cell migration assay

The migration ability of treated RKO and HCT-116 cells were evaluated by using Transwell chambers (Coring, USA). In brief, log phase-treated cells were suspended in FBS-free media at a concentration of 5 × 10^5^ cells/ml. Cells prepared in 500 μl of FBS-free media were plated in the upper wells, and the bottom chambers maintained suitable media with 20 % FBS as a chemoattractant stimulus for 48 h. Migrated cells on the bottom surface of the well were fixed and stained by 0.1 % crystal violet. The stained cells were counted under a microscope in four randomly selected files at a magnification of 200 × .

### Tumorigenesis in nude mice

RKO cells (sh-KPNA2 treated or control cells; 1 × 10^7^ cells/ml) were injected subcutaneously into the left (control cells) and right (sh-KPNA2 cells) flanks region of four nude mice (6-week-old) respectively. The nude mice were purchased from the Institute of Zoology, Chinese Academy of Sciences of Shanghai. After injection, the mice were housed until the tumor was visible. Two weeks later, the mice were sacrificed and the tumors developed from each mouse removed and weighed. All animal protocols were approved by Shanghai Jiaotong University Affiliated Shanghai First People’s Hospital Animal Care.

### Statistical analysis

All data are presented as mean ± SD unless specified otherwise. The differences between the two groups were compared with the *t*-test, *χ*^*2*^ tests, or Fisher’s exact test, as appropriate. The survival rate was analyzed using the Kaplan-Meier method with the log-rank test. In addition, univariate and multivariate Cox-regression analyses were applied to evaluate the hazard ratio considering the KPNA2 expression levels and subjects’ characteristics. All statistical analyses were performed using the SPSS 19.0 software (SPSS Inc, Chicago, IL). A *p*-value <0.05 was considered statistically significant.

## Results

### KPNA2 expression is significantly upregulated in human colon cancer

**Fig. 1:**
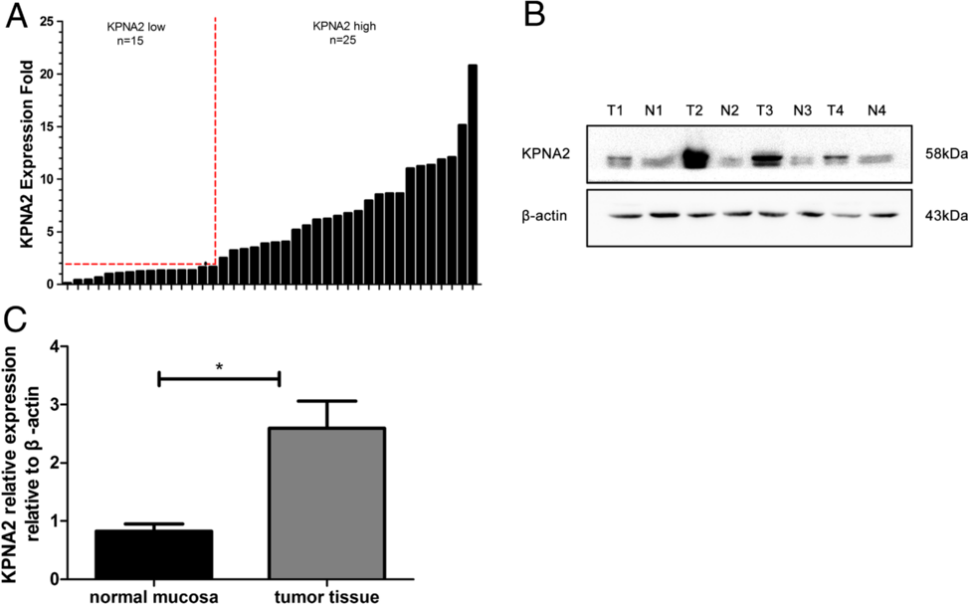
Analysis of KPNA2 expression in human colon cancer tissues. **a** Real-time quantitative PCR analysis of KPNA2 expression in human colon cancer tissues and paired normal tissues. KPNA2 mRNA level was normalized using GAPDH expression. A logarithmic scale of 2^-ΔΔCT^ was used to represent the fold-change. The dashed line represents a 2-fold change normalized to paired normal tissues. **b** Western blot analysis was performed to assess KPNA2 protein levels in 4 representative cases of colon cancer specimens. **c** KPNA2 protein is higher in tumor tissues than in paired adjacent normal mucosa. ‘N’ represents normal tissue; ‘T’ represents tumor tissue. ‘*’ *p* < 0.001

We analyzed 40 paired colon cancer tissues and its adjacent normal tissues to investigate the mRNA expression pattern of KPNA2. Among the 40 paired samples, 25 (62 %, KPNA2) colon cancer patients showed at least a 2-fold increase in the gene mRNA level compared with that in adjacent normal mucosa. In addition, 18 (45 %) colon cancers showed a 5-fold up-regulation in KPNA2 mRNA level corresponding to normal tissues (Fig. 1a). The average KPNA2 expression (ΔCt value) in the colon tumor group was 4.96 ± 0.42 whereas in the normal tissue group, it was 8.65 ± 0.57 (*p* < 0.01). Western blot analysis showed a significant up-regulation of KPNA2 protein expression in cancerous colon tissue compared with that of the corresponding normal tissue (2.60 ± 0.46 vs. 0.83 ± 0.12, respectively; *p* < 0.01, Student’s *t*-test) (Fig. 1b and c).

### Correlation between KPNA2 overexpression and colon cancer clinicopathologic parameters

**Fig. 2:**
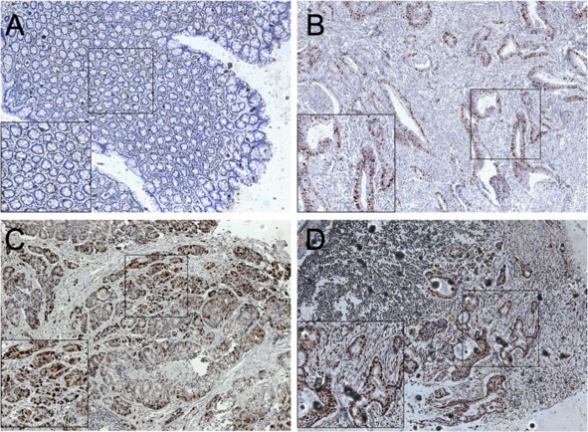
Immunohistochemical staining for KPNA2 in normal and malignant colon tissues. **a**. Negative KPNA2 expression in normal colonic epithelium; **b**. Weak KPNA2 staining in a well-differentiated colon tumor; **c**. Diffuse, intense KPNA2 staining in moderate to poorly differentiated colon tumor; **d**. Strong KPNA2 staining in a colon cancer lymph node metastatic sample. Original magnification × 100

Immunolocalization of KPNA2 protein in the 195 primary colon tumors, as well as paired normal colon mucosa and 66 lymph node metastasis specimens in TMA. We observed that KPNA2 was predominantly stained positively in the nuclei of the primary and lymph node-infiltrated tumor cells, whereas KPNA2 staining was minimally detectable in adjacent normal colon cells (Fig. 2).

Among the 195 samples on the paired TMA, 99 (50.7 %) showed negative staining in adjacent normal mucosa. In contrast, KPNA2 positive staining in primary tumor was significantly higher, with positive staining in 136 (69.7 %) and negative staining in 59 (30.3 %) specimens. It was remarkable that 47 of the 66 (71.2 %) LNM samples also exhibited KPNA2 positive staining. Statistical analysis the KPNA2 expression between primary tumor and paired normal tissues, showed a significant difference between the two groups (*p* < 0.001). But the difference between primary tumor and LNM tissues is not statistically significant (*p* = 0.14) (Table 1).

**Table 1:** KPNA2 immunohistochemical staining

KPNA2 expression					
Tissue sample	*n*	Negative	Weak	Positive	*p*-value
Normal mucosa	195	99 (50.7)	74 (37.9)	22 (11.3)	
tumor	195	59 (30.3)	83 (42.6)	53 (27.2)	<0.001*
LNM	66	19 (28.8)	21 (31.8)	26 (39.4)	0.14

*indicates statistical difference, *p*-value is based on the chi-square test

Table 2 summarizes the relationship between KPNA2 expression levels and a range of clinicopathologic features. KPNA2 over-expression was significantly correlated with the AJCC stage (*p* = 0.01), T-classification (*p* = 0.018), regional lymph node metastasis (*p* = 0.025), distant metastasis (*p* = 0.014), and differentiated degree (*p* = 0.001), while no relationship was found between KPNA2 expression and age, gender, tumor location or vessel invasion status. We also observed a significant correlation between KPNA2 and presence of Ki-67 (*p* = 0.002), suggesting a potential role for KPNA2 in the promotion of cancer cell proliferation and growth.

**Table 2:** Association between clinicopathologic features and KPNA2 protein expression

KPNA2 expression					
	*n*	Negative	Weak	Positive	*p*-value
Age (years)					0.746
< 65	78	22 (28.2)	36 (46.2)	20(25.6)	
≥ 65	117	37 (31.6)	47 (40.2)	33 (28.2)	
Sex					0.665
Male	83	25 (30.1)	33 (39.8)	25 (30.1)	
Female	112	34 (30.4)	50 (44.6)	28 (25.0)	
Location					0.664
Right	82	21(25.6)	37 (45.1)	24 (29.3)	
Transverse	18	9 (50.0)	7 (38.9)	2 (11.1)	
Descendent	18	5 (27.8)	7(38.9)	6 (33.3)	
Left	77	25(32.5)	32 (41.6)	20 (26.0)	
AJCC stage					0.01*
I	22	12 (54.5)	7 (31.8)	3 (13.6)	
II	79	25 (31.6)	37 (46.8)	17 (21.5)	
III	76	21 (27.6)	30 (39.5)	25 (32.9)	
IV	18	1 (5.6)	8 (44.4)	9 (50.0)	
N stage					0.025*
N0	104	40 (38.5)	45 (43.3)	19 (18.3)	
N1	57	14 (24.6)	22 (38.6)	21 (36.8)	
N2	34	6 (17.6)	15 (44.1)	13 (38.2)	
M stage					0.014*
M0	177	58 (32.8)	75 (42.4)	44 (24.9)	
M1	18	1 (5.6)	8 (44.4)	9 (50.0)	
T stage					0.018*
T_1_ + T_2_	28	14 (50.0)	12 (42.9)	2 (7.1)	
T_3_ + T_4_	167	46 (27.5)	71 (42.5)	50 (30.0)	
Differentiation					0.001*
High	97	39 (40.2)	41 (42.3)	17 (17.5)	
Moderate	68	13 (19.1)	33 (48.5)	22 (32.4)	
Low	30	8 (26.7)	8 (26.7)	14 (46.7)	
Vascular invasion					0.604
Yes	14	3 (21.4)	8 (57.1)	3 (21.4)	
No	181	56 (30.9)	75 (41.4)	50 (27.6)	
Ki-67 expression					0.002*
Negative	36	11 (30.6)	22 (61.1)	3 (8.3)	
Positive	159	48 (30.2)	60 (37.8)	50 (31.4)	

*p < 0.005 indicates a significant association between the variables

### High KPNA2 expression associated with poor clinical outcome in human colon cancer

**Fig. 3:**
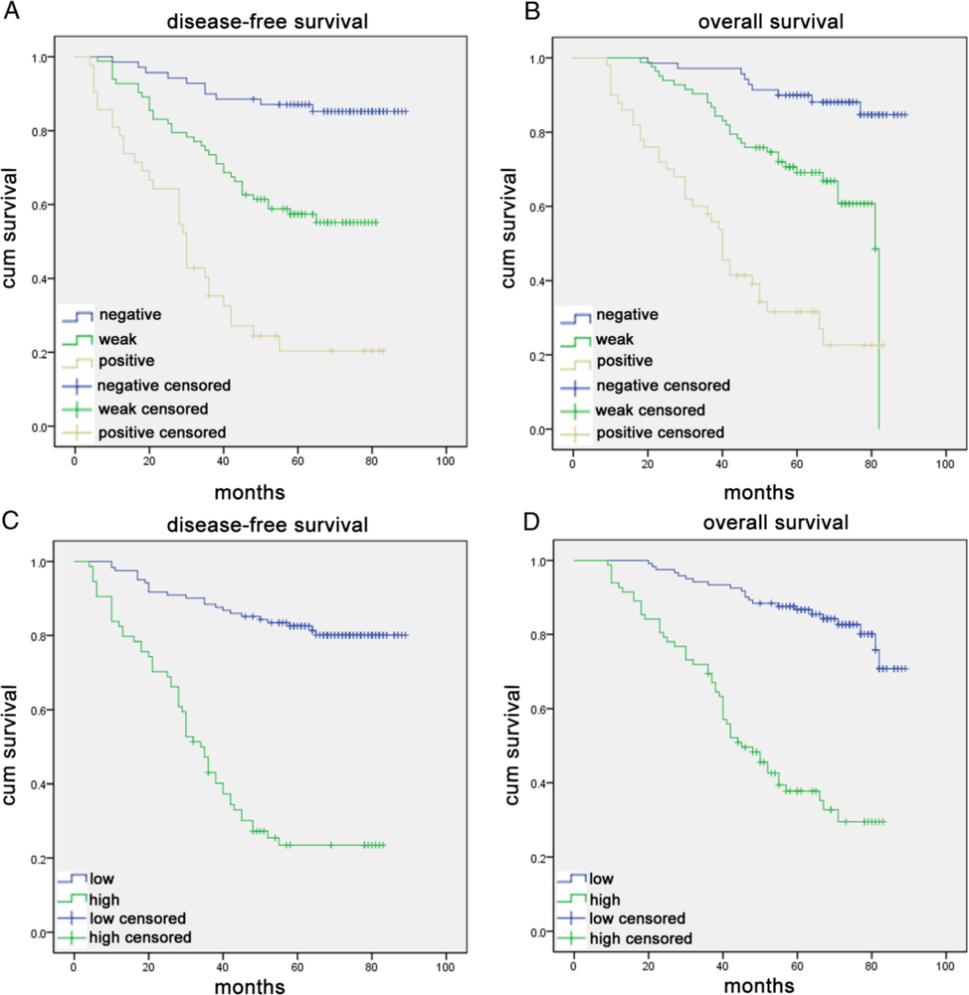
Kaplan-Meier analysis with a log-rank test of survival. **a** and **c** The disease-free survival (DFS) and **b** and **d** 5-year overall survival (OS) of patients were associated with KPNA2 expression that was determined by immunohistochemical staining. DFS and OS were significantly shorter in patients with KPNA2 high expression tumors than in those with KPNA2 low expression tumors

To test the predictive role of KPNA2 for distant metastasis, KPNA2 staining was analyzed with the survival data and examined by Kaplan Meier survival analysis. Of the 195 patients, 8 patients had undergone non-curative surgery were excluded in the calculation of DFS. There was a significant difference between KPNA2-positive and KPNA2-negative groups after the surgery (Fig. 3). Patients with KPNA2 positive staining showed poor prognosis, and accrued no benefit in recurrence [KPNA2-positive: 65 of 128 patients (50.7 %); KPNA2-negative: 8 of 59 (13.6 %); (*p* < 0.001)], meanwhile the 5-year OS (*p* < 0.001) and DFS (*p* < 0.001) were remarkably lower than those patients with KPNA2-negative stained tumors (Fig. 3).

In univariate analysis, pN stage, pM stage, AJCC stage, and vascular invasion were correlated with OS and DFS (Table 3). We conducted a multivariate analysis using the Cox proportional hazard model for all the significant variables in univariate analysis, and the expression of KPNA2 was found to be an independent prognostic indicator to predict tumor recurrence (Table 4). A significant association was found between the high KPNA2 expression level and lower 5-year DFS (HR = 1.681, 95 % CI: 1.170–2.416, *p* = 0.005) and OS survival (HR = 2.770, 95 % CI: 1.314–5.83, *p* = 0.007) (Table 4).

**Table 3:** Univariate analysis of the DFS and OS in 195 colon cancer patients

Variable	DFS			OS		
	HR	95 % CI	*p*	HR	95 % CI	*p*
Age (years)						
< 65						
> 65	0.958	0.581–1.547	0.832	1.048	0.581–1.888	0.877
Sex						
male						
female	0.959	0.593–1.552	0.865	1.227	0.681–2.212	0.496
Location						
right						
others	1.143	0.957–1.365	0.139	1.159	0.936–1.436	0.176
AJCC stage						
I + II						
III + IV	3.248	2.256–4.675	<0.001*	15.219	4.281–54.102	<0.001*
T stage						
T_1_ + T_2_						
T_3_ + T_4_	1.638	1.160–2.313	0.005*	1.226	0.846–1.777	0.282
N stage						
N0						
N1 + N2	3.293	2.413–4.494	<0.001*	2.569	1.773–3.724	<0.001*
M stage						
M0						
M1	5.641	2.661–11.958	<0.001*	8.713	3.786–20.055	<0.001*
Differentiation						
Well + moderate						
Poor	1.404	1.010–1.952	0.043	1.363	0.915–2.032	0.128
Vascular invasion						
no						
Yes	4.356	2.266–8.35	<0.001*	3.705	1.646–8.337	0.002*
Ki–67 expression						
low						
high	1.311	0.952–1.084	0.097	0.924	0.659–1.295	0.646
KPNA2 expression						
low						
high	1.969	1.410–2.748	<0.001*	1.729	1.158–2.583	0.007*

*HR* hazard ratio, *CI* confidence interval. *p < 0.005 indicates that the lower limit of the 95 % CI of HR is >1

**Table 4:** Multivariate analysis of the DFS and OS in 195 colon cancer patients

Variable	DFS			OS		
	HR	95 % CI	*p*	HR	95 % CI	*p*
AJCC stage						
(I/II vs. III/IV)	2.669	1.491–4.779	0.001*	9.030	1.198–68.048	0.033*
N stage	2.890	1.696–4.926	<0.001*	2.528	1.177–5.426	0.017*
M stage	4.261	1.225–14.820	0.023*	5.711	1.586–20.568	0.008*
Vascular invasion	1.946	0.955–3.963	0.067	1.685	0.687–4.130	0.254
KPNA2 expression	1.681	1.170–2.416	0.005*	2.770	1.314–5.837	0.007*

*HR* hazard ration, *CI* confidence interval. *p < 0.005 indicates that the lower limit of the 95 % CI of HR is >1

### Inhibition of colon cancer cell growth by sh-RNA induced down-regulation of KPNA2 expression

**Fig. 4:**
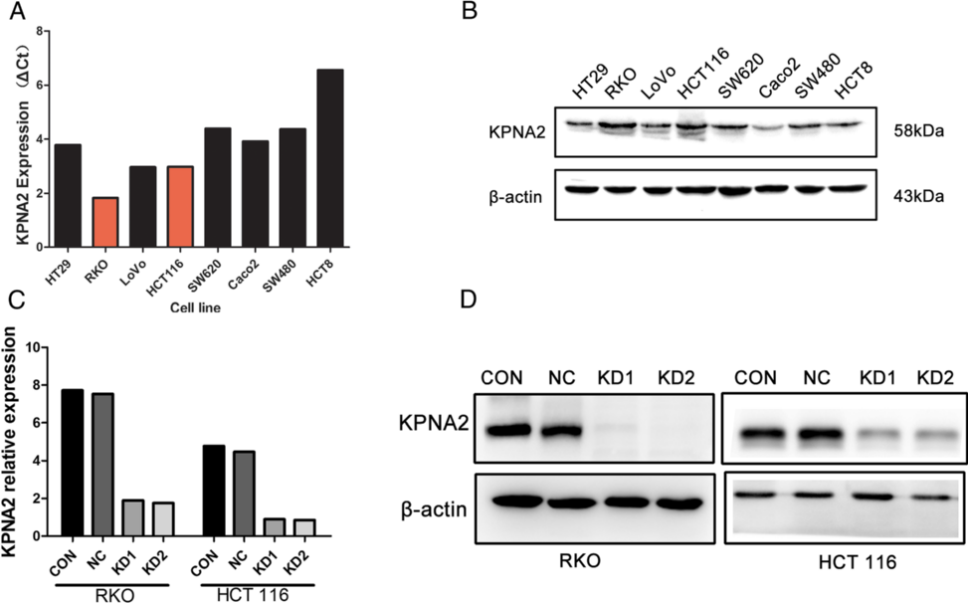
KPNA2 expression in cell lines. **a** KPNA2 mRNA levels and **b** protein levels in colon cancer cell lines; KPNA2 mRNA and protein expression in RKO and HCT-116 treated with KPNA2 shRNA1- and shRNA2- were validated using **c** real-time PCR, and **d** Western blotting. CON: blank control group; NC: negative control group; KD1: KPNA2 shRNA1; KD2: KPNA2 shRNA2

To test the role of KPNA2 on colon cancer, RNA interference (RNAi) technology was carried out to access the role of KPNA2 in colon cancer cells. KPNA2 expression level was higher in RKO and HCT-116 than other cell lines (Fig. 4a, b). We confirmed the efficacy of knockdown of KPNA2 expression using RT-PCR (Fig. 4c) and Western blotting (Fig. 4d).

**Fig. 5:**
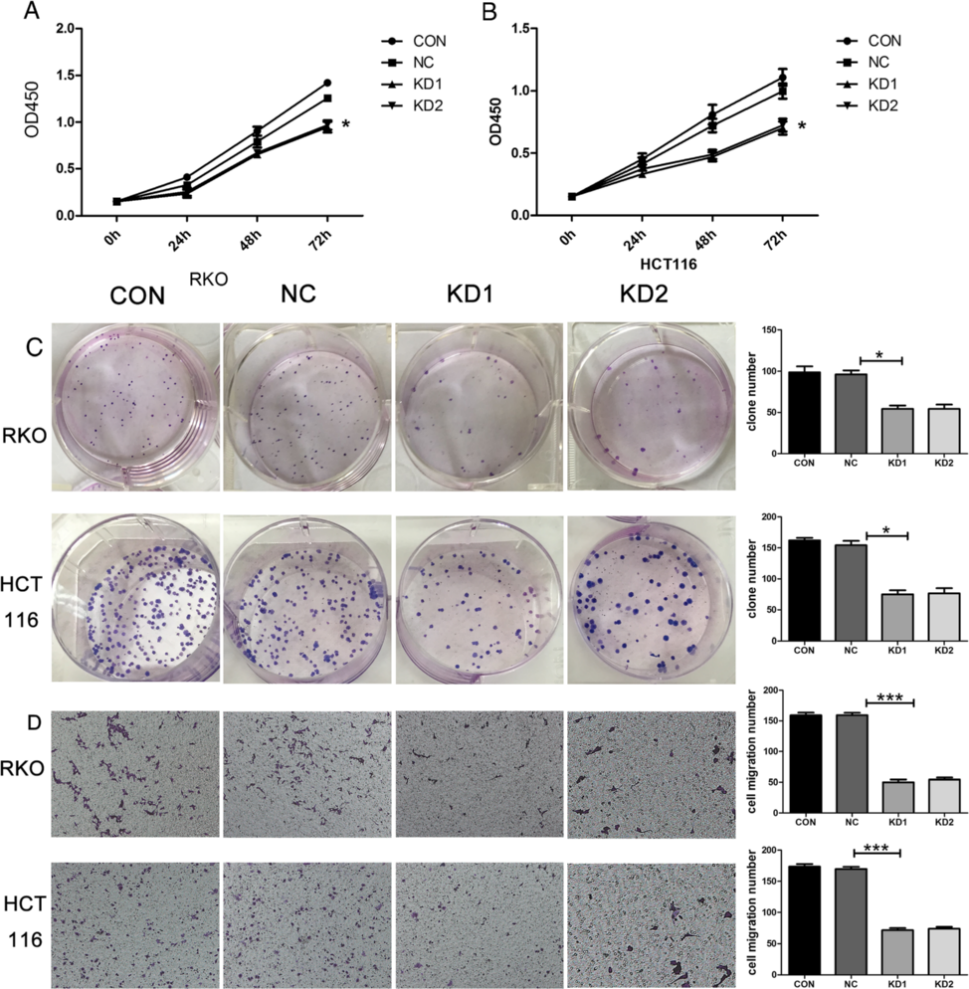
Functional analysis of human colon cancer cell lines treated with KPNA2 shRNA. KPNA2 knockdown inhibits colon cancer cell **a**-**b** growth (*P < 0.05), **c** colony formation (*P < 0.05) and **d** invasion (***P < 0.01). CON: blank control group; NC: negative control group; KD1: KPNA2 shRNA1; KD2: KPNA2 shRNA2

A CCK-8 assay showed that knockdown of KPNA2 expression significantly decreased survival and proliferation in contrast with control cells (*p* < 0.001, Fig. 5a, b). Similarly, silencing of KPNA2 reduced the number of colony formation (*p* < 0.001, Fig. 5c). This result was consistent with the finding that increased KPNA2 expression has a direct correlation with a high proliferation index in colon tumor specimens. These data suggested that up regulated KPNA2 contributes to survival and proliferation of colon cancer cells.

### Attenuation of the migratory ability of colon cancer cells by KPNA2 silencing

Because KPNA2 expression was correlated with both nodal and distant metastasis of colon cancer (Table 2), we investigated the role of KPNA2 in the migration of colon tumors. By applying Transwell Boyden chamber assay, we found that compared with negative control cells, cells lacking KPNA2 displayed reduced migration (Fig. 5d). The results support the hypothesis that KPNA2 is an important biomarker for migration of colon cancer cells.

### KPNA2 expression is necessary for in vivo tumor size growth

**Fig. 6:**
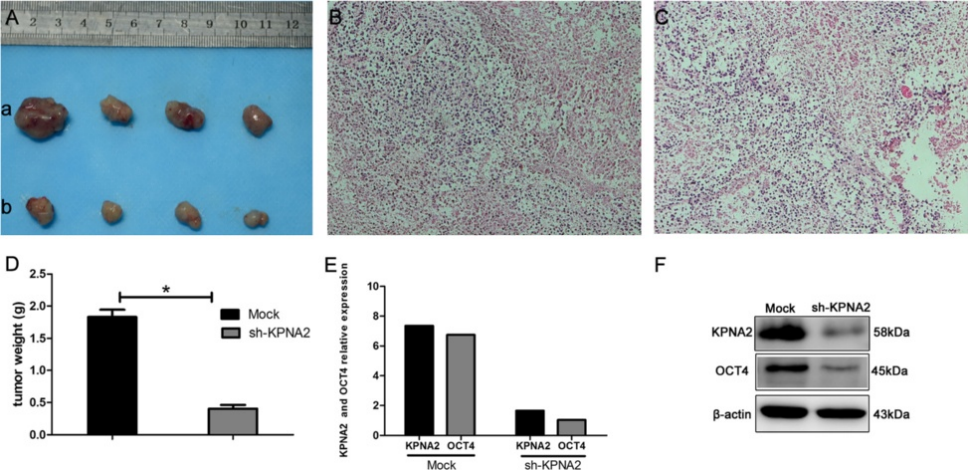
Effect of KPNA2 knock down on nude mice transplantation tumor of RKO cells. RKO cells with mock transfection (*a*) or KPNA2-shRNA1 (*b*) were implanted into the flanks region of four nude mice. **a** After tumor development, the animals were sacrificed and tumors were removed. **b** The HE staining of xenograft tumors from (a Mock group). **c** The HE staining of xenograft tumors from (b sh-KPNA2 group). **d** The tumors from different groups were weighted. **e** Expression of KPNA2 and OCT4 mRNA were detected in Mock and sh-KPNA2 groups by Real-time quantitative PCR. **f** KPNA2 and OCT4 protein expression in Mock and sh-KPNA2 groups were detected by western blot. KPNA2 and OCT4 expression were suppressed in sh-KPNA2 group

To investigate the effects of decreased KPNA2 expression on colon tumor growth, we “knocked down” KPNA2 expression in RKO clones by stably transfecting the cells with KPNA2 shRNA. The treated RKO cells with decreased KPNA2 along with RKO control cells were injected subcutaneously into the left (mock cells) and right (KPNA2-shRNA cells) flanks region of four nude mice respectively. After tumor development, the animals were sacrificed and tumors were removed and weighed (Fig. 6a, d). We found that the tumors with reduced levels of KPNA2 were significantly smaller and lighter than those that RKO control cells. The HE staining of xenograft tumors were shown (Fig. 6b, c).

OCT4 is a homeodomain transcription factor of the POU family, a cargo protein of the KPNA2. We examined the expression of OCT4 and KPNA2 in the tumors. The expression of OCT4 in the nucleus was suppressed in the KPNA2 shRNA group compared with the control group (Fig. 6e, f).

## Discussion

Dysfunction of the cellular transport machinery is common in cancer [5]. The nuclear transport signaling pathway was previously identified as important for tumorigenesis and tumor development in several types of cancer [10]. KPNA2 is an adaptor protein that mediates the import of signaling factor into the nucleus and export of response molecules to the cytoplasm [16]. KPNA2 is expressed more in various cancers than in normal tissues, and high expression of KPNA2 has been confirmed as a predictor of poor prognosis in different cancers [7, 8, 11, 12, 17–19]. In this study, we found that KPNA2 expression is elevated in human colon cancer, and that its aberrant expression is tied to an adverse outcome in the patients. We also found a positive association between KPNA2 expression and advanced tumor stage, indicating that KPNA2 might play a critical role in the progression of colon cancer. These are consistent with previous Rachidi’s study, they have found a drastic increase in KPNA2 expression in primary and lymph node metastatic colon tumors compared to adjacent normal tissues, KPNA2-positive cells increased from T1 through T4 [11].

Studies have indicated that aberrant KPNA2 expression can be found in early lesions, such as ductal carcinoma in situ (DSIS) in breast cancer [20] and non-invasive bladder cancer samples [21]. In our research statistical analysis, the KPNA2 expression in primary tumor and LNM tissues, showed no significant difference between the two groups. These findings are important, as they represent opportunities to gain prognostic information at an early stage.

The Ki-67 antigen is a cell proliferation marker; Ki-67 expression strictly correlates with cell cycle progression and may be observed in G_1_-, S_1_-, and G_2_-phase and mitotic cells [22]. In the present study, Ki-67 was selected to represent the proliferation status of the cells. We observed a significant correlation between KPNA2 expression and the presence of Ki-67. Studies have shown that KPNA2 appears to play a critical role in arranging cellular proliferation, tumor invasion, and aggressive behavior [7, 17, 18, 20, 21]. The present data also suggested a potential role of KPNA2 in the promotion of colon cancer cell proliferation and growth.

A reasonable way by which KPNA2 could affect carcinogenesis is through the translocation of cancer-associated cargo proteins. Several cargo proteins of KPNA2 have been identified to date, including NBS1, OCT4, NFKB1, Myc, p53, LEF-1, CHK2, BRCA1, S100A_2_, S100A_6_, RAC1 and p65 [23]. OCT4 has been reported to be an oncogenic fate determinant. High levels of OCT4 increase the malignant potential of embryonic stem derived tumors [24]. Researchers have found that miR-26b/KPNA2/OCT4 axis inhibited epithelial ovarian cancer cells viability, migratory ability in vitro and in vivo. KPNA2 was validated as a direct target of miR-26b. Knockdown of KPNA2 or ectopic expression of miR-26b could downregulate OCT4 [25]. Xiao-Lei Li also found that reduction of KPNA2 expression significantly reduced mRNA and nucleoprotein levels of OCT4 [26]. We examined the expression of OCT4 in xenograft tumors, and found that mRNA and nucleus protein levels of OCT4 were significantly downregulated in KPNA2 knockdown group. In our study, knock down of KPNA2 decreased the proliferation, colony formation, and migration activity of colon cancer cells, giving further credit to the idea that KPNA2 affects the viability of cancer cells. Therefore, KPNA2 appears to be a major determinant of the subcellular localization and biological functions of its cargo proteins. It is plausible that KPNA2 functions as a pleiotropic modulator of colon cancer progression, although the molecular mechanisms need to be further elucidated.

Study limitations include the small scale of investigated patients with relatively short follow-up time, lack of overexpressed cancer cell lines. The molecular mechanisms of KPNA2 deregulation in colon cancer need to be further studied.

## Conclusions

In summary, the present study identified that the expression of KPNA2 mRNA and protein was upregulated in colon tumor samples in comparison to that in normal tissues. KPNA2 overexpression correlates with poor survival in large colon cancer patient cohorts. KPNA2 expression could be a useful prognostic marker for colon cancer patients. In addition, KPNA2 regulated colon cancer cell proliferation, colony formation, and migration. In animal experiments, the in vivo tumorigenicity was weakened effectively by downregulation of KPNA2. This spurs the idea that KPNA2 may hold an important role in the carcinogenesis of colon cancer.
